# There Is a Differential Pattern in the Fatty Acid Profile in Children with CD Compared to Children with UC

**DOI:** 10.3390/jcm11092365

**Published:** 2022-04-23

**Authors:** Justyna Kikut, Arleta Drozd, Małgorzata Mokrzycka, Urszula Grzybowska-Chlebowczyk, Maciej Ziętek, Małgorzata Szczuko

**Affiliations:** 1 Department of Human Nutrition and Metabolomics, Pomeranian Medical University in Szczecin, 71-460 Szczecin, Poland; justyna.kikut@pum.edu.pl (J.K.); arleta.drozd@pum.edu.pl (A.D.); 2 Department of Pediatrics, Hemato-Oncology and Pediatric Gastroenterology, Independent Public Clinical Hospital No. 1, Pomeranian Medical University in Szczecin, 71-252 Szczecin, Poland; malgorzata.mokrzycka@pum.edu.pl; 3 Department of Pediatrics, Faculty of Medical Sciences in Katowice, Medical University of Silesia in Katowice, Medyków 16, 40-752 Katowice, Poland; uchlebowczyk@sum.edu.pl; 4 Department of Perinatology, Obstetrics and Gynecology, Pomeranian Medical University in Szczecin, 72-010 Police, Poland; maciej.zietek@pum.edu.pl

**Keywords:** Crohn’s disease, ulcerative colitis, inflammatory bowel disease, adolescents, fatty acids, vaccenic acid, gamma linoleic acid, linoleic acid, palmitic acid, lauric acid

## Abstract

Background: Crohn’s disease (CD) and Ulcerative Colitis (UC) are classified as inflammatory bowel diseases (IBD). Currently, an increasing number of studies indicate that the metabolic consequences of IBD may include abnormalities in the fatty acid profile. The aim of this study was to compare fatty acid concentrations in IBD in order to identify differences between CD and UC and differences between the phases of both diseases. Methods: Sixty-three adolescent patients with CD (*n* = 33) and UC (*n* = 30) aged 13.66 ± 2.67 and 14.15 ± 3.31, respectively, were enrolled in the study. Analysis was performed by gas chromatography. Results: A statistically significant higher concentration of vaccenic acid was observed in the total UC group relative to total CD. In remission CD relative to active CD, a significantly higher concentration of palmitic acid was shown. Whereas in active CD, significantly higher levels of linoleic acid were observed relative to remission. The UC group had significantly higher lauric acid and gamma-linoleic acid levels in active disease relative to remission. Conclusions: The identified differences between FA levels in UC and CD could potentially be involved in the course of both diseases.

## 1. Introduction

Crohn’s disease (CD) and Ulcerative Colitis (UC) are classified as inflammatory bowel diseases (IBD). Clinically, CD is characterized by multiform intestinal trans wall inflammation complicated by abscesses and fistulas. Additionally, in the case of CD, the entire gastrointestinal tract may be affected. In contrast, in UC, a neutrophilic inflammation is observed and the disease itself is limited to the colon [1,2]. The pathogenesis of IBD is not completely known, but there are indications that IBD is associated with environmental, genetic, and immunologic factors as well as changes in the gut microbiome [3]. The incidence and prevalence of IBD are not only recorded in Europe, but are already observed globally [4]. While IBD prevalence is still highest in Western countries, the incidence appears to be stabilizing in this region [4]. Unfortunately, an increase in incidence is also observed among the pediatric population and it is estimated that around 25% of patients with IBD are under 20 years of age [2]. Currently, pharmacological treatment of IBD is based on the use of corticosteroids, amino salicylates, immunosuppressants and antibiotics and supportive treatment [5], and pharmacotherapy involves reducing and controlling the inflammatory process [6].

Fatty acids are composed of hydrocarbon chains containing a carboxyl group and a methyl group. Their biological activity depends not only on the chain length, but also on the presence and number of double bonds. Unsaturated fatty acids are characterized by at least one double bond, while saturated fatty acids contain no double bond [7]. Polyunsaturated fatty acids (PUFAs) are distinguished by two or more double bonds [7]. Fatty acids (FA) can be divided into short-chain fatty acids (SCFA), i.e., those with up to 5 carbon atoms, medium-chain fatty acids (MCFA) containing 6 to 12 carbon atoms and long-chain fatty acids (LCFA) with 13 to 20 carbon atoms [8]. Lipids are responsible for the integrity and fluidity of the cell membrane, intercellular signaling and for the production of eicosanoids as well. Disruption of these functions significantly affects cell homeostasis and the spread of inflammation, and thus may lead to the development of IBD [8]. In IBD, inflammatory cell infiltration such as T lymphocytes, macrophages, mast cells or plasma cells is observed. Currently, more and more studies indicate that the metabolic consequences of IBD may include disturbances in the profile of fatty acids and their pro-inflammatory mediators [9]. In contrast, long-chain fatty acids may influence the regulation and quieting of inflammation [10]. A study by Naito et al. showed that oral administration of arachidonic acid (AA) to rats with inflammatory bowel disease increased inflammatory reaction in contrast to healthy animals [11]. On the other hand, omega 3 fatty acids such as EPA and DHA influenced the reduction in proinflammatory eicosanoids, regulating the production of cytokines, which reduced the occurrence of inflammation [9]. It appears that the identification and modification of eicosanoids and their precursors may show clinical benefits in the treatment of IBD [9].

The aim of this study was to compare fatty acid concentrations in IBD in order to identify differences between CD and UC. The inclusion of both disease entities, together with the division into activity phases, will allow differences in the course of inflammation to be illustrated and will improve knowledge regarding the introduction of new therapeutic strategies leading to IBD remission.

## 2. Material and Methods

The study received positive permission from the Bioethics Committee of the Pomeranian Medical University (KB-0012/131/18 dated 26 November 2018). Written informed consent to participate in a research study was obtained from the parents or legal guardians of the patients. In addition, patients over 16 years of age gave written consent to participate in the study. In addition, as the participation was voluntary, all participants were free to decline to participate at any time of the study.

### 2.1. Study Group

**Figure 1:**
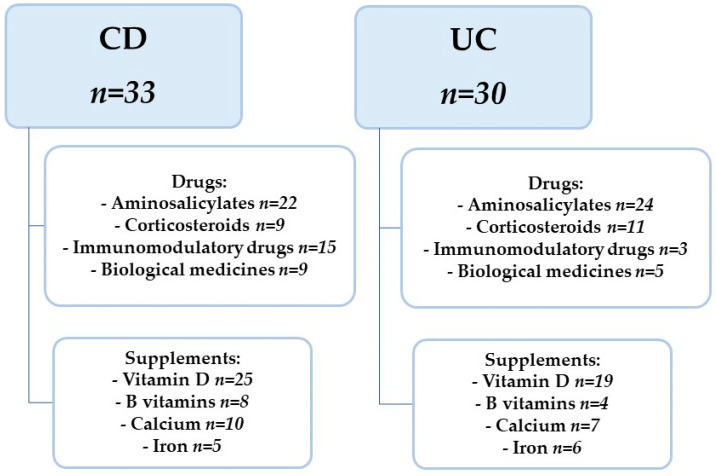
Characteristics of drugs and dietary supplements used. CD—Crohn’s Disease, UC—Ulcerative Colitis.

A group of 63 patients with a diagnosis of Crohn’s disease or Ulcerative Colitis was eligible to participate in the study. There were 33 patients in the CD group (15 girls, 18 boys) and 30 patients in the UC group (13 girls, 17 boys). Patient characteristics are presented in Table 1. Patients were enrolled in the study during their stay in the gastroenterology departments of two university hospitals. IBD was diagnosed based on endoscopic examination (Olympus, Tokyo, Japan) with subsequent histopathological evaluation. Upper and lower gastrointestinal endoscopy were performed in patients with the diagnosis. Additionally, in patients with a diagnosis of CD, an ultrasound scan, computed tomographic (CT) enterography and enteroclysis were performed. A diagnosis of CD or UC and an age range of 7–18 years were used as research study inclusion criteria. Exclusion criteria were parenteral nutrition implemented and the use of specialized elimination diets. Referring to the phase of the diseases, their activity was assessed using questionnaires for CD Pediatric Crohn’s Disease Activity Index (PCDAI) and for UC Pediatric Ulcerative Colitis Activity Index (PUCAI). There were 23 and 24 subjects in CD and UC, respectively, in exacerbation of the disease. In contrast, 10 subjects in CD and 6 subjects in UC were in remission. In addition, in the CD group, 19 patients were diagnosed up to one year time from diagnosis to examination, while in UC this was 21 patients. The remaining patients had a diagnosis of more than one year from the time of diagnosis. Characteristics of drugs and dietary supplements are shown in Figure 1. In addition, antibiotic therapy was used only in those patients who had current indications for it, i.e., during exacerbation with high inflammatory parameters.

### 2.2. Anthropometric Measurements

Bodyweight (±0.1 kg) and height (±0.5 cm) measurements were collected to determine differences in the nutritional status of the patients. A medical scale (Radwag WPT 60/150 OW, Radom, Poland) with a height meter was used to obtain accurate results. The obtained results were compared to the centile grids for body weight and BMI of OLA and OLAF recommended by the Institute “Pomnik—Centrum Zdrowia Dziecka” (Warsaw, Poland) [12].

### 2.3. Sample Collection

Venous blood (7 mL) was collected from patients into a tube on an EDTA medium. The whole blood was then centrifuged at 3500 rpm for 10 min. The obtained plasma and morphotic elements were separated into separate eppendrophs (500 µL). The material thus prepared was stored in a freezer at −80 °C until analysis, which was performed using plasma by gas chromatography (GC).

### 2.4. Extraction and Quantification of Fatty Acids

The analysis was performed on an Agilent Technologies 7890A GC with a SUPELCOWAX 10 Capillary column using the Folch method with minor modifications [13]. The results were analyzed using ChemStation Software (Agilent Technologies, Cheadle, UK). The retention time of the fatty acids analyzed was compared with Food Industry FAME Mix (cat. No 35077) (Restek, Bellefonte, PA, USA), which was supplemented with the following standards: C18:4 (stearidonic acid methyl ester, cat. No 10005000), C22:4n6 (docosatetraenoic acid methyl ester, cat. no 10006866) and C22:5n3 (docosapentaenoic acid methyl ester, cat. no 21124, Cayman Chemical) by using ChemStation Sofware (Agilent Technologies, Cheadle, UK). C21:0 (heneicosaenoic acid, Merck, Tokyo, Japan) was used as a fatty acid retention time control. The results are presented as the percentage of each fatty acid to the total fatty acid mass from the tested sample. The exact methodology of the study was described in a previous article [14,15].

### 2.5. Statistical Analysis

Statistica 13.3 software (Statsoft, Krakow, Poland) was used to perform statistical analysis. Normality of data distribution was checked using Shapiro–Wilk test. The data presented a normal distribution. Comparison of the active phase of the patients was performed using the parametric T-student test. On the other hand, the analysis of the active phase and remission and the comparison of both phases of each disease were performed using the non-parametric Mann–Whitney U test. A *p*-value < 0.05 was reported as statistically significant.

## 3. Results

Fatty acids such as 4:0 Butyric acid, C6:0 Caproic acid, C8:0 Caprylic acid, C11:0 Undecanoic acid, C15:1 cis-10-pentadecanoid acid, C18:2n6t Linoleic acid (trans LA), C18:4 stearidonic acid (SDA), C20: 3n3 Eicosatrienoic acid (ETE), C22:0 Behenic acid, C22:1n9/13 (Erucic acid), C22:2 cis-docodienoic acid, C23:0 tricosanoic acid, C24:0 Lignoceric acid and C24:1 Nervonic acid were analyzed in the samples, but they were not determinable.

A comparison of the percentage of FA in both disease entities (CD and UC) showed a statistically significantly higher content of one acid, vaccenic acid C18:1 (*p* = 0.035) (Table 2).

Comparing active phase vs. remission CD, there was a statistically significant higher C16:0 palmitic acid concentration (*p* = 0.047) in remission CD and a statistically significant higher C18:2n6c linoleic acid (cis LA) concentration in active CD (*p* = 0.025) (Table 3).

When comparing the active phase vs. remission of UC, a statistically significant higher concentration of C12:0 Lauric acid was observed in active UC (*p* = 0.041). C18:3n6 gamma-linoleic acid (GLA) concentration was only determinable in patients with active UC; hence, statistical significance (*p* = 0.031) (Table 3).

A comparison of the active and remission phase of both diseases has shown no statistically significant differences (Table 3).

## 4. Discussion

It seems important to understand the involvement of FA and their mediators in the course of remission and exacerbation of both diseases. Understanding the mechanisms of FA release from cell membranes in IBD may help to develop new therapeutic strategies, especially since AA and LA derivatives act as epigenetic modulators [16]. Although the involvement of fatty acids in the course of IBD has been studied, their role remains unclear and debatable and thus this study is precursory. To our knowledge, few studies have focused on the fatty acid characterization in pediatric patients with IBD [17,18,19]. Moreover, most studies do not compare the two diseases with respect to the phase of their activity.

In the present study, we reported no significant differences in SFA, MUFA and PUFA levels either among the two disease entities or within the two disease phases. Similarly, a study by Socha et al., which included a pediatric group in a similar age range, comparing PUFA, MUFA and SFA ratios, showed that PUFA profiles were similar in the CD and UC groups [17]. However, in this study, only the active phase of the disease was analyzed. In contrast, as is observed by Esteve-Comas et al., higher levels of n-3 fatty acids in the active phase of the disease may be related to increased biosynthesis, but also to a higher intake of n-3 fatty acids [20]. In addition, lower n-3 levels were not observed in active disease compared to a control group [20].

In our study, differences were observed considering CD group and UC group in total vaccenic acid levels. Similarly, although no significant differences were observed between the relevant phases of the two disease entities in this study, they were observed between phases within the disease entity. Vaccenic acid (VA) represents one of the most common naturally occurring trans acids in the human food chain. Studies in rats confirm its beneficial effects on the accumulation and breakdown of hepatic adipose tissue, indicating its hypolipidemic effects and protective role in metabolic syndrome [21]. Other studies also point to the role of vaccenic and oleic acids as good predictors for focal liver lesions [22]. The potential role of VA in inflammatory bowel disease has also been highlighted. Supplementation of VA in rats followed by an analysis of the jejunal mucosa demonstrated a potential alternative therapeutic pathway for inflammatory bowel disease [23]. In the mentioned study, the addition of VA to the diet of rats was shown to inhibit the expression of fatty acid amide hydrolase (FAAH) in the intestine, which, in turn, may activate anti-inflammatory protective pathways of the endocannabinoid system (ECS) in the intestine [23]. With respect to our results, this indicates a possible role for VA in UC. Furthermore, in a study by Hengstermann et al. evaluating fatty acid content between active and inactive IBD, no differences in fatty acid profile were observed between the two phases of the disease [24]. In contrast, in a study by Esteve-Comas et al., the investigators found that C18:3n3 and C22:6n3 acids did not differ from a healthy control group even in the severe form of the disease. The authors suggest that this may be due to the fact of increased biosynthesis co-occurring with an increased intake of PUFA, which, in turn, at the time (1992), questioned the validity of using these acids in the treatment of IBD [20]. Similar to the study of Esteve-Comas et al., we observed a similar percentage of palmitic acid in the blood of subjects with active UC and CD, but in CD remission, its concentration was lower, which may indicate increased elongation to AA acid of its cascade. Interestingly, the concentration in the UC remission group was comparable to the control group in the cited study [20].

The level of palmitic acid (C16:0) differed significantly in the CD group; there was an increase in palmitoleic acid (C16:1) and a chain elongation to stearic acid (C18:0). The latter then desaturates to its metabolites such as oleic acid (C18:1w9) and vaccenic acid (C18:1 trans11). This synthesis pathway using sterol regulatory element-binding transcription factor 1 (SREBP-1c) is stimulated by the high insulin and cholesterol levels that accompany the metabolic syndrome [14]. In vitro studies have shown that palmitic acid affects the integrity and permeability of the intestinal epithelial barrier, particularly in conditions where intestinal permeability is impaired. Palmitic acid promotes increased levels of inflammatory cytokines by activating pro-inflammatory pathways through receptors including TLR4 [25,26,27]. Although the exact mechanism of activation of the palmitic acid pathway is not well understood, it is suspected that changes in the gut microbiota may contribute [27]. This, in turn, points to the risk of increased intestinal permeability in patients with CD at a higher rate than UC. Other studies have indicated palmitic acid-induced impairment of insulin signaling in human Caco2/TC7 enterocytes and the involvement of PA in insulin resistance, thus contributing to metabolic diseases [28]. This raises the possibility that people with CD in adulthood may be more predisposed to metabolic syndrome than patients with UC. Similarly, in a study by Levy et al. involving pediatric patients with CD regardless of disease stage, statistically significant higher plasma levels of palmitic acid C16:0 and C18:2n6 were observed in CD patients relative to controls [18]. A study by Ito et al., where FA were isolated by the same method, showed statistically significant differences in C16:0 palmitic acid and C18:2n6 linoleic acid levels in relation to the control group only [29]. Another study suggests palmitoleic acid (PO) as a potential biomarker that may reflect the severity of CD, as its levels were correlated with disease activity and C-reactive protein (CRP) levels [30]. In our study, we observed statistically significant higher LA levels in active CD relative to remission. In contrast, Scoville et al. observed no differences in serum LA levels between adults with CD and controls [31]. In opposition to the results of our study, higher LA levels were observed in adults in inactive disease relative to active disease and, in general, higher LA levels relative to the present study [32]. Similar observations were made in the pediatric group in the study by Trebble et al. [19]. The differences may be due to many factors such as variations in the number and age of participants involved in the study, genetic differences, treatment modalities, diagnostic process and accurate diagnosis.

A statistically significant higher concentration of C12:0 Lauric acid was observed in active UC relative to remission, which indicates the activation of elongation for the synthesis of longer chain fatty acids and desaturation to MUFA. Interestingly, lauric acid is the only one in the MCFA group to be absorbed by a similar mechanism to LCFA. This may also explain the fact that the other MCFA tested were not detected in quantifiable amounts. Additionally, C12:0 is an antagonist for the immune system, stimulating toll-like receptor 4 (TLR4) protein and forming monolaurin in the gut, which in turn exerts antimicrobial effects [33]. In another study, lauric acid in a dose-dependent manner induced activation of the nuclear transcription factor NF kappa B (NF-κB) and expression of interleukin 8 (IL-8) [34]. Perhaps lauric acid could be a part of UC therapy. Interestingly, in a further analysis of our study, we observed that GLA for UC is only labeled in the active phase of the disease. ALA is a substrate for GLA, which is then elongated to C20:3n6 eicosatrienoic acid and finally to C20:4n6 arachidonic acid [35]. It can be suggested that lower ALA levels may be a consequence of GLA synthesis, which may influence the downstream inflammatory cascade within the inflammatory AA metabolites, the eicosanoids. Perhaps GLA could be a marker to determine UC activity. Furthermore, it is worth noting that γ-linolenic acid (GLA, C18:3n-6) is the only member of the n-6 fatty acid family to exhibit anti-tumor activity [36,37,38]. Some studies identify GLA as potentially mitigating and/or inhibiting inflammation and having therapeutic benefits; however, results regarding possible supplementation are inconclusive [39,40]. In a study involving adult patients with UC, it was observed that 8-week supplementation with cis-Palmitoleic acid reduced inflammatory activity in patients with active UC by decreasing the expression of the pro-inflammatory interleukin-6 in the patients’ mucosa [41].

A limitation of the study was the small number of studies conducted to date in the pediatric population. Thus, the minor differences in fatty acid concentrations in the current study compared to other studies may be because the vast majority of studies are based on adults. Limitations of the study may also be the diversity of the study group and the lack of a control group. Nevertheless, this article, in relation to the small number of studies on this topic, points to a new target for future research.

## 5. Summary

In our study, we observed the involvement of vaccenic acid in the course of UC, in general, as other studies have also shown. Perhaps further studies can confirm the involvement of vaccenic acid as a potential therapeutic pathway. Analyzing the different phases of UC, only in the active phase of the disease is GLA determined, which may be analyzed in further studies as a potential marker to differentiate UC activity, and it may explain the involvement of lauric acid in the active phase of the disease. In turn, palmitic acid may be involved in remission in CD, whereas linoleic acid is involved in the active phase of the disease. This indicates a higher risk of increased intestinal permeability in patients with CD than UC, and a greater predisposition to metabolic syndrome. Certainly, further research into the potential use of individual fatty acids in alternative therapies for inflammatory bowel disease is worthwhile. In conclusion, the identified differences between FA levels in UC and CD could potentially be involved in the course of both diseases.

## Figures and Tables

**Table 1:** Characteristics of patients with CD and UC.

Parameter	CD *n* = 33		UC *n* = 30		*p*-Value
	Girls *n* = 15	Boys *n* = 18	Girls *n* = 13	Boys *n* = 17	
Age (years)	13.66 ± 2.67		14.15 ± 3.31		0.522
Body mass (kg)	46.29 ± 18.1		53.02 ± 19.4		0.159
Height (m)	1.54 ± 0.19		1.60 ± 0.2		0.218
BMI percentile	42.16 ± 35.39		47.03 ± 37.74		0.531
Body mass percentile	38.05 ± 34.52		46.40 ± 37.50		0.246
PCDAI	16.04 ± 16.33		-		-
PUCAI	-		30 ± 23.36		-
Duration of disease (months)	27.71 ± 31.25		30.04 ± 23.36		-
Fecal calprotectin active disease (uq/g)	2606.68 ± 2504.64		2230.07 ± 2113.7		0.66
Fecal calprotectin (uq/g)	2040.45 ± 2269.53		2096.77 ± 2110.49		0.94

CD—Crohn’s Disease, UC—Ulcerative Colitis, PCDAI—Pediatric Crohn’s Disease Activity Index, PUCAI—Pediatric Ulcerative Colitis Activity Index. BMI—Body Mass Index

**Table 2:** Characteristics of fatty acids in CD and UC groups.

FA%	Avg ± SD CD *n* = 34	Avg ± SD UC *n* = 30	*p*-Value
C12:0 Lauric acid	0.141 ± 0.101	0.141 ± 0.049	0.984
C13:0 Tridecanoic acid	0.599 ± 0.179	0.600 ± 0.158	0.982
C14:0 Myristic acid	1.056 ± 0.215	1.059 ± 0.213	0.951
C14:1 Myristolenic acid	0.056 ± 0.050	0.046 ± 0.056	0.465
C15:0 Pentadecanoid acid	0.274 ± 0.043	0.256 ± 0.044	0.115
C16:0 Palmitic acid	25.932 ± 1.641	26.814 ± 2.402	0.092
C16:1 Palmitoleic acid	0.790 ± 0.513	0.862 ± 0.565	0.597
C17:0 Heptadecanoid acid	0.580 ± 0.116	0.549 ± 0.127	0.309
C17:1 cis-10- Heptadecanoid acid	0.154 ± 0.100	0.165 ± 0.122	0.702
C18:0 Stearic acid	27.810 ± 3.621	27.461 ± 3.176	0.687
C18:1n9 ct Oleic acid	12.919 ± 2.298	12.692 ± 2.106	0.685
C18:1 vaccenic acid	1.224 ± 0.194	1.349 ± 0.264	0.035
C18:2n6c Linoleic acid (cis LA)	10.054 ± 2.167	9.363 ± 2.239	0.218
C18:3n6 gamma linoleic acid (GLA)	0.706 ± 2.069	1.432 ± 2.768	0.240
C18:3n3 linolenic acid (ALA)	0.230 ± 0.118	0.190 ± 0.120	0.192
C20:0 Arachidic acid	0.471 ± 0.174	0.426 ± 0.209	0.362
C22:1/C20:1 cis11- eicosanic acid	0.259 ± 0.119	0.221 ± 0.123	0.214
C20:2 cis-11-eicodienoic acid	0.075 ± 0.092	0.051 ± 0.089	0.293
C20:3n6 dihomo-gamma-linolenic acid (DGLA)	1.264 ± 0.293	1.243 ± 0.279	0.772
C20:4n6 Arachidonic acid (AA)	9.340 ± 1.611	9.191 ± 1.749	0.726
C20:5n3 Eicosapentaenoic acid (EPA)	0.505 ± 0.560	0.448 ± 0.148	0.587
C22:4n6 docosatetraenoic acid (DTA)	1.673 ± 0.406	1.644 ± 0.357	0.770
C22:5w3 Docosapentaenoic acid (DPA)	1.376 ± 0.376	1.447 ± 0.312	0.420
C22:6n3 Docosahexaenoic acid (DHA)	2.512 ± 0.950	2.349 ± 0.857	0.479
Total SFA	55.410 ± 14.432	55.901 ± 14.573	0.850
Total MUFA	15.248 ± 5.536	15.170 ± 5.424	0.970
Total PUFA n6	23.112 ± 4.565	22.924 ± 4.263	0.977
Total PUFA n3	4.623 ± 1.028	4.434 ± 0.989	0.958

CD—Crohn’s disease; UC—Ulcerative colitis; FA—fatty acids, SFA—saturated fatty acids; MUFA—monounsaturated fatty acids, PUFA—polyunsaturated fatty acids.

**Table 3:** Characteristics of fatty acids in CD and UC patients in active phase and remission (%).

Fatty Acids (%)	CD Active	CD Remission	*p*-Value CD Active vs. CD Remission	UC Active	UC Remission	*p*-Value UC Active vs. UC Remission	*p*-Value CD vs. UC Active	*p*-Value CD vs. UC Remission
C12:0 Lauric acid	0.123 ± 0.118	0.149 ± 0.035	1.00	0.150 ± 0.048	0.108 ± 0.040	0.041	0.970	0.357
C16:0 Palmitic acid	25.383 ± 1.811	26.17 ± 1.038	0.047	27.17 ± 2.514	25.402 ± 1.177	0.082	0.128	0.957
C18:2n6c (cis LA)	11.364 ± 1.868	9.485 ± 2.332	0.025	9.150 ± 2.126	10.213 ± 2.681	0.484	0.570	0.303
C18:3n6 (GLA)	0.889 ± 1.824	0.627 ± 2.651	0.897	1.790 ± 2.999	0.000 ± 0.000	0.031	0.117	0.357
C13:0 Tridecanoic acid	0.549 ± 0.177	0.621 ± 0.182	0.269	0.579 ± 0.152	0.686 ± 0.165	0.140	0.379	0.093
C14:0 Myristic acid	0.999 ± 0.220	1.081 ± 0.202	0.185	1.063 ± 0.219	1.044 ± 0.207	0.897	0.786	0.786
C14:1 Myristolenic acid	0.044 ± 0.051	0.061 ± 0.047	0.439	0.045 ± 0.056	0.051 ± 0.059	0.795	0.307	0.745
C15:0 Pentadecanoid acid	0.277 ± 0.044	0.272 ± 0.044	0.713	0.257 ± 0.046	0.254 ± 0.038	0.856	0.244	0.416
C16:1 Palmitoleic acid	0.725 ± 0.559	0.818 ± 0.408	0.768	0.935 ± 0.602	0.571 ± 0.239	0.288	0.494	0.871
C17:0 Heptadecanoid acid	0.568 ± 0.114	0.585 ± 0.124	0.581	0.531 ± 0.120	0.617 ± 0.142	0.288	0.123	0.481
C17:1 cis-10- Heptadecanoid acid	0.150 ± 0.100	0.156 ± 0.106	0.825	0.165 ± 0.128	0.164 ± 0.104	0.897	0.785	0.871
C18:0 Stearic acid	26.983 ± 3.486	28.17 ± 3.977	0.686	27.08 ± 3.064	28.994 ± 3.435	0.223	0.260	0.175
C18:1n9 ct Oleic acid	12.323 ± 2.602	13.18 ± 1.286	0.686	12.92 ± 2.275	11.787 ± 0.841	0.364	0.717	0.551
C18:1 vaccenic acid	1.208 ± 0.192	1.230 ± 0.208	0.462	1.352 ± 0.267	1.338 ± 0.279	0.776	0.081	0.212
C18:3n3 (ALA)	0.254 ± 0.111	0.219 ± 0.134	0.450	0.183 ± 0.123	0.218 ± 0.112	0.517	0.300	0.828
C20:0 Arachidic acid	0.451 ± 0.176	0.479 ± 0.176	0.439	0.397 ± 0.220	0.546 ± 0.095	0.052	0.162	0.175
C22:1/C20:1 cis11- eicosanic acid	0.235 ± 0.129	0.270 ± 0.091	0.556	0.208 ± 0.133	0.271 ± 0.057	0.422	0.117	0.551
C20:2 cis-11-eicodienoic acid	0.113 ± 0.085	0.059 ± 0.101	0.185	0.049 ± 0.089	0.060 ± 0.093	0.697	0.695	0.416
C20:3n6 (DGLA)	1.342 ± 0.267	1.230 ± 0.348	0.632	1.199 ± 0.239	1.417 ± 0.377	0.140	0.680	0.625
C20:4n6 (AA)	9.777 ± 1.646	9.151 ± 1.516	0.377	9.009 ± 1.782	9.918 ± 1.532	0.337	0.779	0.786
C20:5n3 (EPA)	0.421 ± 0.670	0.542 ± 0.091	0.508	0.450 ± 0.139	0.438 ± 0.193	0.517	0.515	0.481
C22:4n6 (DTA)	1.744 ± 0.434	1.642 ± 0.342	0.286	1.585 ± 0.337	1.881 ± 0.366	0.102	0.619	0.551
C22:5w3 (DPA)	1.408 ± 0.435	1.362 ± 0.195	0.198	1.444 ± 0.346	1.459 ± 0.118	0.736	0.477	0.481
C22:6n3 (DHA)	2.669 ± 1.030	2.444 ± 0.760	0.162	2.295 ± 0.913	2.564 ± 0.600	0.452	0.603	0.704
Total SFA	53.936 ± 14.07	56.05 ± 14.59	0.676	55.854 ± 14.566	56.09 ± 14.65	1.00	0.676	1.000
Total MUFA	14.535 ± 5.283	15.56 ± 5.646	0.676	15.458 ± 5.519	14.02 ± 5.045	1.00	0.835	1.000
Total PUFA *n*-6	25.229 ± 4.989	22.19 ± 4.387	0.575	22.783 ± 4.136	23.49 ± 4.822	0.936	0.943	0.810
Total PUFA *n*-3	4.752 ± 1.111	4.567 ± 0.993	0.885	4.373 ± 0.968	4.679 ± 1.075	0.885	0.885	0.885

CD—Crohn’s Disease; UC—Ulcerative Colitis; LA—linoleic acid; GLA—gamma-linoleic acid; ALA—linolenic acid; DGLA—dihomo-gamma-linolenic acid; AA—arachidonic acid; EPA—eicosapentaenoic acid; DTA—docosatetraenoic acid; DPA—docosapentaenoic acid; DHA—docosahexaenoic acid, SFA—saturated fatty acids; MUFA—monounsaturated fatty acids, PUFA—polyunsaturated fatty acids; red color indicates statistically significant results (*p*-value < 0.05)

## Data Availability

Not applicable.
